# Dangerous Measures: A Case Report and Review of Motoro Ray Envenomation

**DOI:** 10.3390/toxins18060270

**Published:** 2026-06-19

**Authors:** Philip Dwek, Omer Jamal, Shaleesa Clarke, Kiran Wadhawan, Andrea K. Boggild

**Affiliations:** 1 Lakeridge Health, Oshawa, ON L1G 2B9, Canada; 2 Institute of Medical Science, University of Toronto, Toronto, ON M5S 1A8, Canada; 3 Temerty Faculty of Medicine, University of Toronto, Toronto, ON M5S 1A8, Canada; 4 Department of Medicine, University of Toronto, Toronto, ON M5S 1A8, Canada; 5 Tropical Disease Unit, Toronto General Hospital/University Health Network, Toronto, ON M5G 2C4, Canada

**Keywords:** Aquatic envenomation, home aquarium, One Health, ornamental fish, venomous animal, stingray, neurotoxin, toxin

## Abstract

Aquatic envenomations may cause severe tissue injury, neurologic morbidity, and even mortality among those whose leisure and/or occupational activities expose them to marine and freshwater animals. The Motoro ray, or *Potamotrygon motoro* (also known as an ocellate river stingray) is endemic to freshwater tributaries throughout Brazil, and is a frequent source of severe envenoming of local fisherman and those residing near waterways. Local wound management including immersion in warm water, wound cleaning and debridement, as well as antibiotics are mainstays of treatment, as are local anesthetics (e.g., nerve blocks) and systemic opioid analgesics; however, high-quality evidence supporting such interventions is lacking. We present a case of a Canadian who was envenomed by his pet Motoro ray, and describe his clinical presentation and evolution of symptoms over the subsequent months. With the ever-increasing trade of exotic wildlife, clinicians, public health authorities, and those within the broader wildlife regulatory ecosystem should be attuned for unanticipated adverse consequences, such as those described herein. We further situate this case within the existing published literature around this particular species of ray, which is not typically considered an ornamental fish.

## 1. Introduction

**Figure 1:**
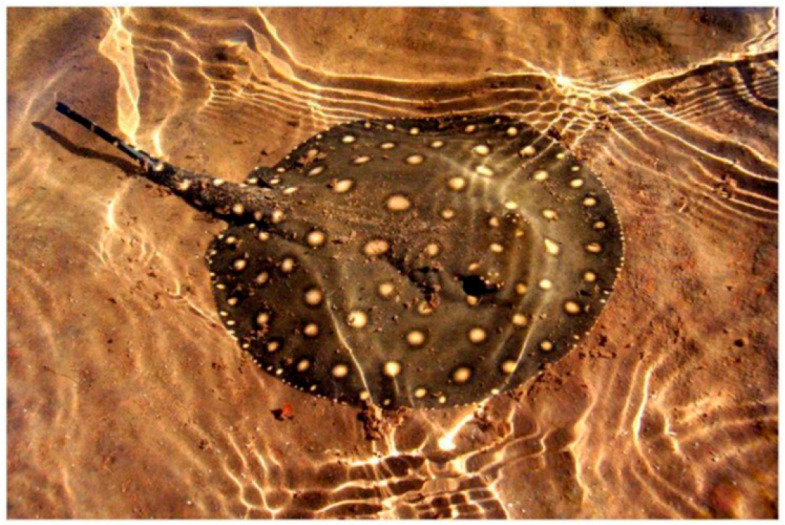
Adult Motoro ray (*Potamotrygon motoro*) from the Araguaia-Tocantins basin of Brazil. Photo courtesy of: Itamar Júnior Tonial (2013). Reproduced from [1] under the creative commons license: https://creativecommons.org/licenses/by/4.0/ (accessed 15 June 2026).

Aquatic envenomations are a common cause of morbidity among those whose leisure and/or occupational activities expose them to marine and freshwater environments. The Motoro ray, or *Potamotrygon motoro* (also known as an ocellate river stingray; Figure 1) is endemic to freshwater tributaries throughout Brazil, and is a common cause of severe envenoming of local fisherman and those residing in riverine communities [1,2]. Following immediate warm water immersion, local wound management including cleaning, debridement, and oral or intravenous antibiotics are the pillars of treatment, as are local anesthetics (e.g., nerve blocks) and systemic opioid analgesics, however high-quality clinical trials supporting these interventions are lacking [1,2]. We present a case of a Canadian who was envenomed by his pet Motoro Ray, and describe his clinical presentation and evolution of symptoms over the subsequent months. Readers will understand the causative species and global epidemiology of marine envenomation syndromes, and also the spectrum of clinical illness manifesting in those envenomed by a marine or aquatic animal.

**Figure 2:**
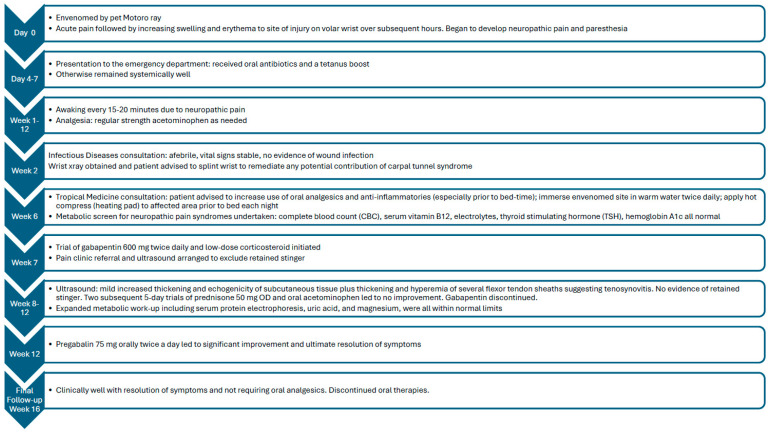
Timeline of symptom evolution and treatment.

**Case Presentation**: A 63-year-old previously well man reported acute onset of percutaneous injury accompanied by pain to the volar wrist after dropping a measuring tape into his tank of pet stingrays. The culprit envenomator was immediately identified as a Motoro ray. In addition to pain at the puncture site, over the subsequent hours and days the patient developed increasing surrounding erythema and swelling, which prompted him to seek medical attention via the emergency department (ED), after which he was treated successfully for a presumed local skin infection with oral antibiotics (Figure 2). At his initial medical visit, his tetanus immunization history was reviewed and he was provided a booster dose of Tetanus–diphtheria (Td) toxoid. He denied associated fever, sweats, or other systemic symptoms in the aftermath of the sting, but shortly after the injury began to note persistent nocturnal numbness below the elbow, and a burning, stinging, prickling sensation radiating down all fingers ipsilateral to the puncture site. These neuropathic symptoms persisted for more than 12 weeks and awoke him from sleep every 15–20 min, and, while not noted to be positional, would resolve with 15 min of walking at the time of occurrence. There was no reported motor weakness or other neurologic symptoms. The patient was taking no medications and had no relevant past medical history. A CT scan of the head obtained previously for investigation of benign paroxysmal positional vertigo was normal.

On examination at the initial Infectious Diseases consultation 10 days after his ED visit, the patient was afebrile with normal vital signs. Skin examination revealed a small healing scar on the volar right wrist, without surrounding erythema, lymphangitic streaking, ulceration, or obvious retained foreign body. Peripheral nerves including the radial cutaneous and ulnar demonstrated no palpable abnormalities, and Phalen’s and Tinel’s tests for carpal tunnel syndrome were negative. Radial and ulnar pulses were strong. Power was 5/5 throughout with normal deep tendon reflexes. Sensory exam was normal, as were gait and cerebellar assessment. A clinical diagnosis of Motoro ray envenomation syndrome was made, and investigations at consultation included wrist X-ray, which was notable for severe osteoarthritis at the first metacarpal joint with cystic changes and flattening and remodeling of the trapezium, which were felt to be unrelated to the envenomation.

The patient was advised to attempt a trial of wrist splitting due to concerns of possible carpal tunnel syndrome, and consultation with the Tropical Medicine service was sought. The patient was then advised to do the following: increase his use of over-the-counter analgesics (i.e., regular strength acetaminophen) including prior to bed-time; immerse the envenomed site in warm water twice daily; and apply a hot compress (heating pad) to the affected area prior to bed each night. Additionally, a short trial of gabapentin 600 mg twice daily and low-dose corticosteroid were initiated to assist with nocturnal symptom management, and both a pain clinic referral and ultrasound to exclude retained stinger were arranged. Finally, a metabolic screen for etiologies that might contribute synergistically to a neuropathic pain syndrome was also undertaken including complete blood count (CBC), serum vitamin B12, electrolytes, thyroid stimulating hormone (TSH), and hemoglobin A1c, all of which were non-contributory.

At subsequent follow-up, the patient reported ongoing nocturnal symptoms. An ultrasound revealed mild increased thickening and echogenicity in the subcutaneous tissue as well as thickening and hyperemia in several flexor tendon sheaths which suggested tenosynovitis. There was no evidence of retained stinger, and expanded metabolic work-up including serum protein electrophoresis, uric acid, and magnesium, were all within normal limits. After a variety of different regimens including high-dose non-steroidal anti-inflammatories, acetaminophen, two trials of prednisone, and gabapentin, the patient was started on pregabalin at 75 mg orally twice a day with significant improvement and ultimate resolution of symptoms over a period of weeks. By four months following the envenomation injury, all symptoms had resolved.

## 2. Discussion

Aquatic envenomations are a common cause of morbidity among those whose leisure and/or occupational activities expose them to marine and freshwater environments. Venomous fish, rays, snails, and jellyfish can all cause envenomation syndromes ranging from mild local reactions at the site of injury to fatal anaphylaxis, cardiorespiratory collapse, and neurologic syndromes, and given the environmental niches such species enjoy, envenomation fatalities have been reported from most tropical and sub-tropical regions [3]. Most envenomations from stingrays are provoked in nature, after a concealed ray sitting on the ocean or river floor is accidentally trodden upon, at which point the envenomee is lashed and impaled by the barbed, dorsal precaudal spine in which venom glands are embedded [2]. Thus, most stingray envenomations occur on the lower extremities, and manifest as local trauma, ulcerative necrosis, secondary skin- and soft-tissue infections, and more rarely, osteomyelitis [1,2,3]. Skin and soft tissue infections due to host flora—such as Group A *Streptococcus* and *Staphylococcus aureus*—as well as aquatic organisms such as *Streptococcus iniae*, *Mycobacterium marinum*, *Vibrio vulnificus*, and *Aeromonas* spp. need to be considered in cases of obvious secondary infection. Deaths due to stingrays have been reported in North America and the Caribbean, islands of the Pacific, as well as Australia and New Zealand [3].

The Motoro ray, or *Potamotrygon motoro* (also known as an ocellate river stingray) is endemic to freshwater tributaries throughout Brazil, and is a frequent source of severe envenoming of local fisherman and those residing in riverine communities [2]. Due to a high rate of ulcerative and necrotic skin lesions arising from such envenomations, the mainstay of immediate management is pain control and prevention of skin necrosis through anti-inflammatories and warm water immersion, given the possible heat lability of toxins and vasoconstrictive action of venom [2]. The standard treatment for acute stingray envenomation is immediate immersion of the site in extremely hot yet tolerable water. Although this intervention may denature and inactivate some venom toxins [2], direct heat-mediated inhibition of venom constituents may be less clinically relevant than direct anti-nociceptive effects of heat [1]. Indeed, studies in which *Potamotrygon* venoms were heated to 56 °C reduced their nociceptive capacity by only about a third compared to body temperature, and similarly reduced their edematogenic capacity by up to a quarter [1]. As such, heat lability and denaturation of toxins alone are likely insufficiently explanatory for why heat immersion effectively alleviates the acute pain and helps mitigate the chronic inflammation and tissue damage that follows. Within the setting of a pet stingray sting in a local residence, as we describe, this immediate action may have reduced the chronic neurological symptoms experienced later and the attendant healthcare costs. Raising awareness of this critical and urgent intervention amongst emergency responders, poison control centers, and the public would benefit individuals who keep exotic venomous stingrays and fish as pets.

After warm water immersion, local wound management including cleaning, debridement, and antibiotics targeting both Gram-positive (e.g., cephalexin, clindamycin) and Gram-negative (e.g., ciprofloxacin) organisms are used routinely, as are local anesthetics (e.g., nerve blocks) and systemic opioid analgesics [2]. Unlike specific snake envenomings, for which there are robust data to support administration of antivenom [4], no such therapeutic intervention exists or is indicated for Motoro Ray envenomation. Tetanus immunization status should be reviewed and a Td booster administered if not up to date. Highly nociceptive venoms as well as retained stinger parts may contribute to chronic regional neuropathic pain syndromes that typically resolve over several months. Anxiety and impaired function due to insomnia may be profound, both of which may be improved somewhat by anxiolytics, cognitive behavioral therapy, and sleep hygiene techniques, though high-quality evidence to support such interventions is lacking [1]. In Brazil, negative pressure wound therapy (NPWT) along with standard wound care has been successfully used to treat the significant skin and soft-tissue trauma that may result from fresh water stingray envenomings [5].

The Motoro ray is not typically thought of as an ornamental or “pet” fish. However, sophistication, innovation and precision in the entire aquaria ecosystem has enabled enthusiasts to maintain exotic species in captivity and meet their basic physiological needs. The ornamental fish trade represents a major component of the global exotic wildlife industry, with an estimated market value of nearly 5.4 billion US dollars in 2021 [6]. Closely linked to the widespread hobby of aquarium keeping, the sector supports the annual movement of approximately 1.3 billion fish and contributes an estimated 15–20 billion US dollars to the global economy [7]. The industry is highly globalized, encompassing more than 5000 freshwater and 1800 marine species and involving over 80 exporting countries. Demand is concentrated in major importing regions including the United States, United Kingdom, Germany, France, and China, while key exporting countries include Japan, Indonesia, Singapore, the Netherlands, and Sri Lanka. Within Africa, Kenya is a notable exporter. In 2022, Kenya shipped approximately 448,237 kg of ornamental fish globally [6].

Despite its substantial economic and biological scale, the ornamental fish industry is characterized by variable practices and limited knowledge across the supply chain, which can adversely affect both human and animal welfare [8]. With the ever-increasing trade of exotic wildlife, expansion of the knowledge and equipment needed to cultivate exotic species by amateur home enthusiasts, clinicians, public health authorities, and those within the broader wildlife regulatory ecosystem should be attuned for unanticipated adverse consequences, such as those described herein. Indeed, beyond the aforementioned occupations that place individuals at risk of stingray envenomation, workers in the tropical fish retail sector and commercial aquaria, as well as home aquarists uniquely predispose individuals to envenoming outside of geographic regions where knowledge of local species and clinical management are more widespread [9].

## 3. Conclusions

In conclusion, the Motoro ray, also known as an ocellate river stingray, is found in freshwater tributaries throughout Brazil, and is a frequent source of severe envenoming of those reliant on riverine sources of income (including local fisherman) and those residing in riverine communities. Local wound management including cleaning, debridement, and antibiotics are mainstays of treatment, as are local anesthetics (including nerve blocks) as well as systemic opioid analgesics, however high-quality evidence supporting such interventions is lacking. Given the increasing intersection of human and animal habitats, as well as the increasing trend of maintaining exotic species in captivity, as well as particular human-derived pressure on the waterways in which Motoro rays can be found, a comprehensive One Health approach to mitigating risks and minimizing human–ray interactions is needed.

## Data Availability

The original contributions presented in this study are included in the article. Further inquiries can be directed to the corresponding author.
